# Social Media Content of Idiopathic Pulmonary Fibrosis Groups and Pages on Facebook: Cross-sectional Analysis

**DOI:** 10.2196/24199

**Published:** 2021-05-31

**Authors:** Andrew Kochan, Shaun Ong, Sabina Guler, Kerri A Johannson, Christopher J Ryerson, Gillian C Goobie

**Affiliations:** 1 Division of Cardiology Department of Medicine University of British Columbia Vancouver, BC Canada; 2 Division of Respirology Department of Medicine University of Toronto Toronto, ON Canada; 3 Department of Pulmonary Medicine Bern University Hospital University of Bern Bern Switzerland; 4 Division of Respiratory Medicine Department of Medicine University of Calgary Calgary, AB Canada; 5 Division of Respiratory Medicine Department of Medicine University of British Columbia Vancouver, BC Canada; 6 Centre for Heart Lung Innovation Department of Medicine University of British Columbia Vancouver, BC Canada; 7 Clinician Investigator Program Department of Medicine University of British Columbia Vancouver, BC Canada; 8 Department of Human Genetics Graduate School of Public Health University of Pittsburgh Pittsburgh, PA United States

**Keywords:** interstitial lung disease, idiopathic pulmonary fibrosis, patient education, social media, internet

## Abstract

**Background:**

Patients use Facebook as a resource for medical information. We analyzed posts on idiopathic pulmonary fibrosis (IPF)-related Facebook groups and pages for the presence of guideline content, user engagement, and usefulness.

**Objective:**

The objective of this study was to describe and analyze posts from Facebook groups and pages that primarily focus on IPF-related content.

**Methods:**

Cross-sectional analysis was performed on a single date, identifying Facebook groups and pages resulting from separately searching “IPF” and “idiopathic pulmonary fibrosis.” For inclusion, groups and pages needed to meet either search term and be in English, publicly available, and relevant to IPF. Every 10th post was assessed for general characteristics, source, focus, and user engagement metrics. Posts were analyzed for presence of IPF guideline content, useful scientific information (eg, scientific publications), useful support information (eg, information about support groups), and potentially harmful information.

**Results:**

Eligibility criteria were met by 12 groups and 27 pages, leading to analysis of 523 posts. Of these, 42% contained guideline content, 24% provided useful support, 20% provided useful scientific information, and 5% contained potentially harmful information. The most common post source was nonmedical users (85%). Posts most frequently focused on IPF-related news (29%). Posts containing any guideline content had fewer likes or comments and a higher likelihood of containing potentially harmful content. Posts containing useful supportive information had more likes, shares, and comments.

**Conclusions:**

Facebook contains useful information about IPF, but posts with misinformation and less guideline content have higher user engagement, making them more visible. Identifying ways to help patients with IPF discriminate between useful and harmful information on Facebook and other social media platforms is an important task for health care professionals.

## Introduction

Idiopathic pulmonary fibrosis (IPF) is a progressive fibrotic interstitial lung disease (ILD) of unknown etiology characterized by declining lung function, worsening dyspnea, and a poor prognosis [1]. Prior surveys indicate that patients and caregivers perceive a lack of accessible resources and information regarding IPF despite the availability of consensus guidelines [2]. Many online resources provide information about IPF, but these are frequently biased and inaccurate [3]. For example, YouTube videos focused on IPF often contain incomplete, inaccurate, and potentially harmful information, with high levels of user engagement in videos containing inaccurate information [4]. Given recent controversy surrounding Facebook’s policies on censorship of inaccurate or potentially harmful information, it is an especially prescient time to investigate the accuracy of information disseminated via Facebook as it relates to chronic diseases such as IPF [5].

Social media usage in US adults increased from 5% to 72% of the population between 2005 and 2018 [6]. Social media was initially limited to a younger demographic; however, over 40% of people above the age of 65 now use social media [6]. The most widely used forms of social media are YouTube and Facebook, with 72% and 69% of US adults using these platforms, respectively [6]. People frequently use social media for health advice and support, emphasizing the importance of evaluating content and quality of health-related information on these platforms [7-9]. On Facebook, users can post text, pictures, videos, or links, which can be commented on, reacted to, or shared by other users. Facebook pages enable any individual or organization to create public forums where people can interact. Facebook groups are designed for small-group communication where people can discuss topics of common interest, including discussion of medical conditions [10]. Facebook pages are often created by public figures, organizations, industry, and occasionally independent nonmedical users, but may have a more product- or message-based focus than groups [10]. To date, no study has evaluated the types of information available about IPF on Facebook, and whether this represents a useful or potentially harmful resource for patients with IPF and their families.

The objective of this study was to describe and analyze posts from Facebook groups and pages that primarily focus on IPF-related content. We assessed a variety of post characteristics, user engagement metrics, IPF-related content, and the presence of inaccurate information shared in Facebook posts on these groups and pages. We hypothesized that these posts would often be biased, and would frequently contain inaccurate and potentially harmful information, similar to YouTube and other internet resources (see Table S1 in Multimedia Appendix 1 for a complete list of prespecified hypotheses) [3,4].

## Methods

### Search Strategy and Page or Group Selection

A new Facebook account was created after removing all history and cookies from the web browser (Google Chrome). The terms “IPF” and “idiopathic pulmonary fibrosis” were separately entered in Facebook’s search function on January 4, 2019 to identify IPF-related groups and pages. Exclusion criteria included primary language not English, being a “closed” or “private” group, being a group or page with a focus other than IPF, or being a duplicate result.

For all groups or pages that met eligibility criteria, a single researcher recorded basic features, including group or page name, URL, description of group or page, and number of group members or page likes. Given the high number of individual posts, the same basic features were recorded from every 10th post in the group or page, including presence of an external link and its URL, the posting of any image or video, date of posting, and viewer engagement metrics. Three viewer engagement metrics were recorded for each post: the number of likes, shares, and comments.

### Data Extraction

**Figure 1:**
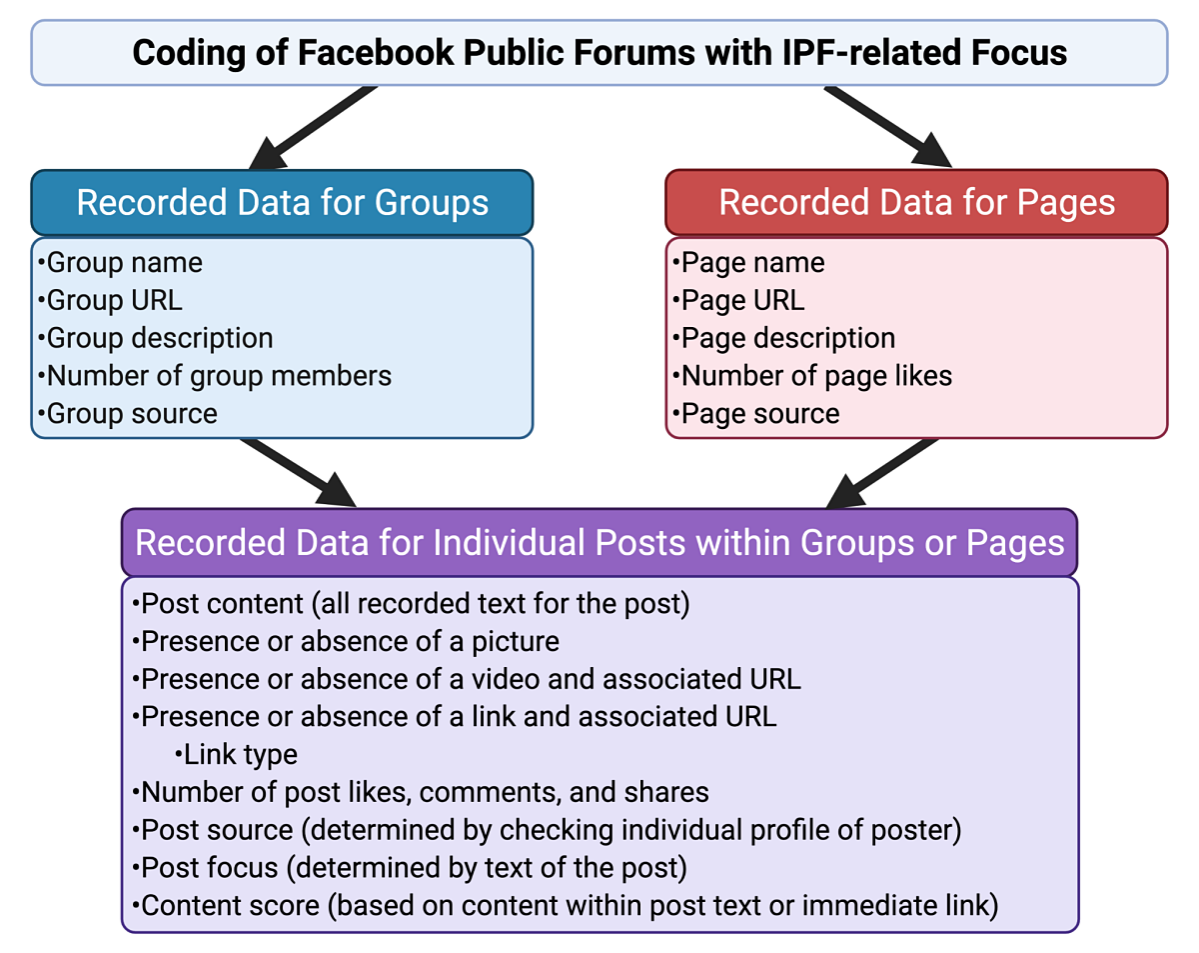
Data extracted from groups, pages, and from individual posts within groups or pages.

IPF-related data were captured in duplicate by two authors who independently reviewed each group or page and post for specific data as detailed in Figure 1. The primary source of the group or page was categorized as scientific resources, medical foundations or organizations, news programs or other media sources, industry or for-profit organizations, private medical professional–generated content, nonmedical user–generated content, or other, as previously described [3]. Individual posts were also separately assigned to one of these sources since the source of a post is not necessarily the same as the source of the group or page. Each post was coded according to the primary focus, including guideline, advice (giving or requesting advice), news (posts about new scientific studies and advances), advertisement or fundraising, opinion (a personal opinion), insurance or health care cost, other IPF content (posts about IPF not falling into a prior category), or non-IPF content (no relation to IPF). If a link was present, the link type was coded as being related to a scientific source, foundation or advocacy, news or media, industry or for profit, or personal blog.

Two authors independently assigned posts a content score based on 30 prespecified guideline-supported IPF-related content items within the categories of definition, symptoms, risk factors, diagnosis, management, and prognosis (Figure 1, Table S2 in Multimedia Appendix 1) [1,11,12]. To assign content scores, authors also evaluated content on the immediate page accessed via any post links. Posts were considered to contain useful scientific information if the post or direct link quoted scientific studies regarding the natural history, diagnosis, or treatment of IPF. Posts were considered to contain useful support information for IPF patients or caregivers if they provided recommendations regarding IPF support networks, peer support, or other practical advice to caregivers or patients with IPF (eg, traveling with IPF, navigating the health care system, strategies for mitigating symptoms). Posts were considered harmful if they recommended pharmacologic or nonpharmacologic therapies (eg, stem cell transplant, specific dietary modifications) not recommended by current IPF guidelines (see Table S3 in Multimedia Appendix 1 for examples of nonrecommended therapies) [1,12].

### Statistical Analysis

Unweighted κ values were used to determine the level of agreement between reviewers for coding of the variables stated above. A κ cut-off value of 0.70 was deemed acceptable. In situations where κ was less than 0.70, a third independent author served as an arbitrator. Descriptive statistics were calculated for overall group and page data, and general post information. Further statistical analysis was performed on individual group and page data as well as combined group and page data. Wilcoxon rank-sum, Spearman correlation, Kruskal-Wallis, and χ^2^ tests were performed as appropriate to analyze potential associations of variables with content score. Wilcoxon rank-sum testing and Fisher exact test were used to identify variables associated with the presence of harmful content, useful scientific information, and useful supportive content.

Zero-inflated negative binomial regression was used to test the association of a higher content score with viewer engagement metrics, post source of foundation or medical professional, and guideline focus. This analysis was adjusted for clustering within groups and pages using a clustered sandwich estimator approach, as posts within individual pages or groups were considered to be dependent on each other [13]. The initial model included all variables considered to have a potential impact on content score followed by elimination of variables with *P*>.05 to achieve a model that met convergence [14]. Multivariable logistic regression was used to identify variables associated with the presence of potentially harmful information within a post. This analysis was also adjusted for clustering within groups or pages using a clustered sandwich estimator approach.

Data are shown as mean (SD), median (IQR), or n (%). Statistical significance was defined by a two-tailed *P*<.05 for all analyses. Analyses were performed using STATA/SE version 14 (StataCorp).

## Results

### Post Characteristics

**Figure 2:**
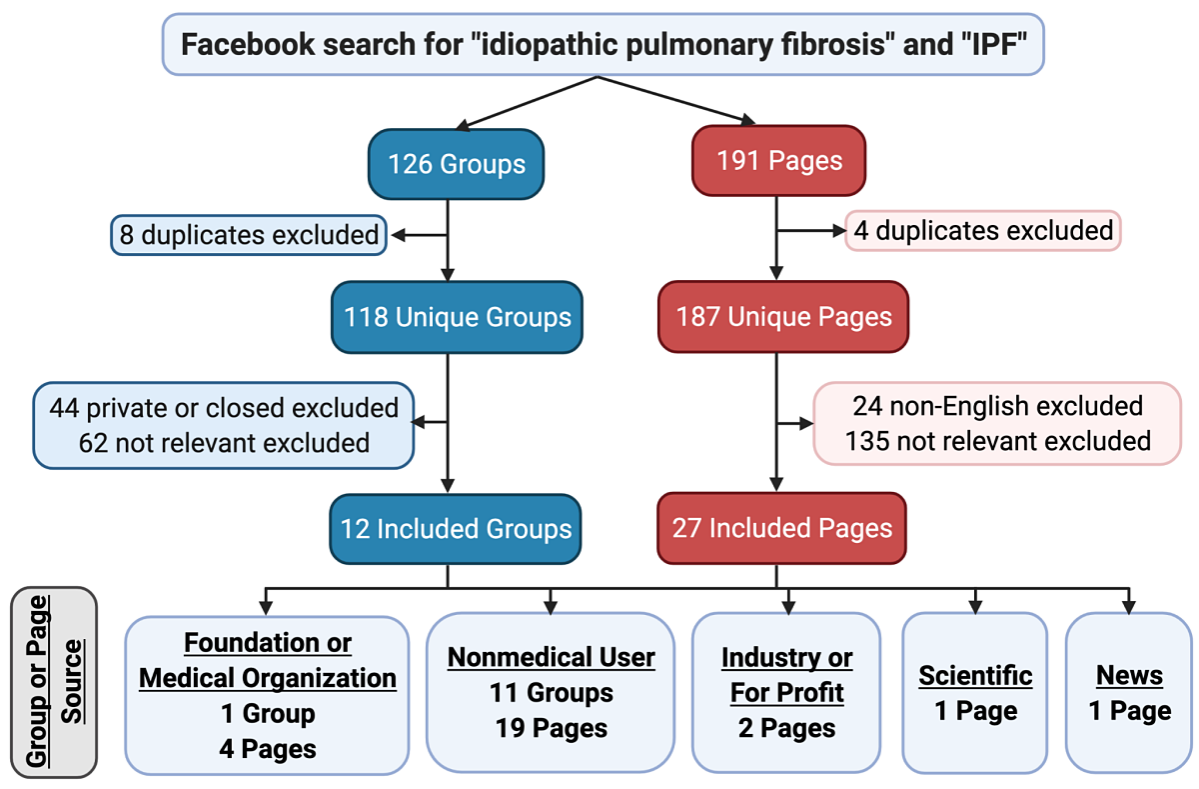
Search results, and selection of groups and pages.

The initial search yielded 126 groups and 191 pages, with 12 groups and 27 pages meeting the eligibility criteria (Figure 2). From the 39 included groups and pages, 523 posts were analyzed. Post source was most frequently from nonmedical users (445/523, 85.1%), followed by foundations or medical organizations (53/523, 10.1%), industry or for-profit organizations (24/523, 4.6%), and private medical professionals (1/523 0.2%). Of the 523 posts analyzed, 307 (58.7%) contained URL links, 118 (22.6%) contained pictures, and 32 (6.1%) contained videos. Median post age was 630 days (IQR 259-1381), with a range from 0 to 4271 days. Viewer engagement, as indicated by the number of likes, comments, or shares, was generally low (Table 1). Post focus was on IPF-related news in 152 posts (29.1%), other IPF-related information in 131 (25.0%) posts, non-IPF commentary in 81 (15.5%) posts, advice to IPF patients or caregivers in 80 (15.3%) posts, advertisement in 65 (12.4%) posts, IPF guidelines in 5 (1.0%) posts, opinion in 7 (1.3%) posts, and insurance in 2 (0.4%) posts. The most common type of external link was to websites presenting IPF-related news (112/523 posts, 21.4%), followed by websites with a foundation or advocacy focus (96/523, 18.4%). Guideline-recommended content was present in 221 (42.3%), useful support in 127 (24.3%), and useful scientific information in 103 (19.7%) of the 523 posts (Figure S1 in Multimedia Appendix 1).

**Table 1:** Baseline characteristics of posts included in study.

Post characteristics	Groups	Pages	Combined
Total posts, N	220	303	523
Picture present, n (%)	32 (14.5)	86 (28.4)	118 (22.6)
Video present (n, %)	5 (2.3)	27 (8.9)	32 (6.1)
Likes per post, mean (SD)	2 (7)	7 (13)	5 (11)
Shares per post, mean (SD)	0 (1)	3 (7)	2 (5)
Comments per post, mean (SD)	1 (3)	1 (3)	1 (3)
Post age (days), median (IQR)	1117 (192-1930)	888 (347-1337)	630 (259-1381)
Link present, n (%)	124 (56.4)	183 (60.4)	307 (58.7)

### Post Content

**Figure 3:**
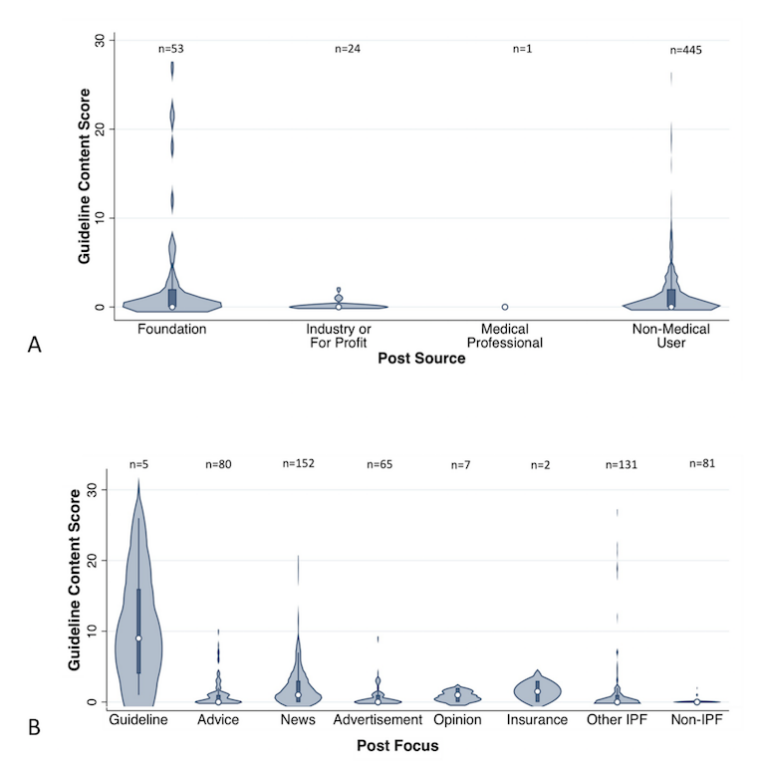
Content score reflecting the number of guideline-recommended content contained in each post or immediate link from groups and pages combined (maximum score=30). The width of the plot at each level corresponds to the number of posts within that group that had that score. Medians with IQRs are presented as a box plot within the violin plot. Posts are delineated by source (A) and focus (B).

We hypothesized that post source of a foundation/medical organization or medical professional, post with a guideline focus, and a post with greater viewer engagement would be associated with higher content scores. On unadjusted analysis, posts from a foundation or medical organization had a numerically higher content score, although this was not statistically significant (Table 2). Posts from an industry source were associated with lower content scores on adjusted analysis (Table 3). Posts with a guideline-related focus had a significantly higher content score (Table 2, Figure 3). On adjusted analysis, post focus on IPF guidelines was associated with lower odds of having a content score of zero, whereas a non-IPF post focus was associated with higher odds of having a content score of zero (Table 3).

**Figure 4:**
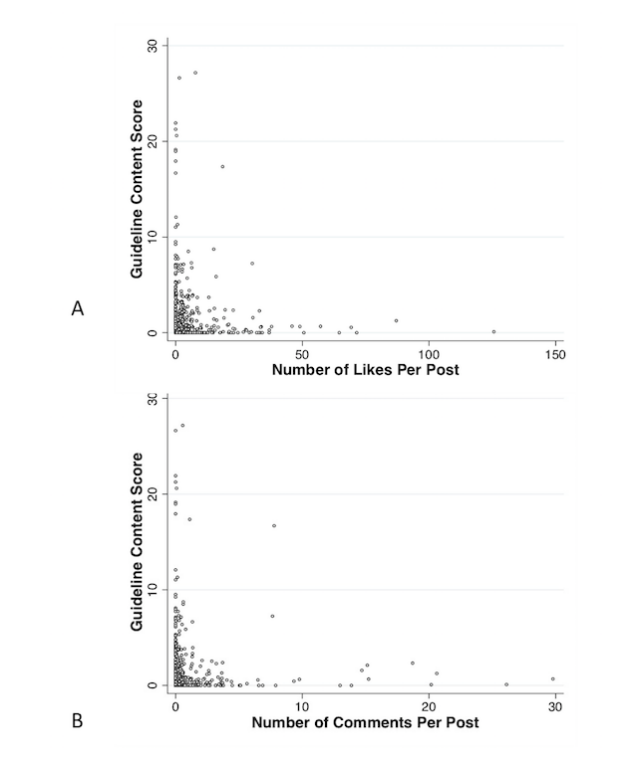
Correlation between number of post likes (A) or number of post comments (B) and content score from groups and pages combined.

With regard to viewer engagement, on unadjusted analyses, there was a negative correlation between number of likes or comments and content score, and posts with a higher number of likes and comments were significantly less likely to contain any guideline-recommended content (Figure 4). There was no correlation between content score and number of shares. Conversely, on adjusted analysis, the number of comments was positively associated with a higher content score (Table 3).

**Table 2:** Mean post content scores broken down by post source and content category from groups and pages combined.

Post variable		Content score^a^, mean (SD)	*P* value^b^
**Source**			.78
	Foundation or medical organization (n=53)	2.8 (6.1)	
	Industry or for profit (n=24)	0.2 (0.5)	
	Medical professional (n=1)	0.0 (0.0)	
	Nonmedical user (n=445)	1.4 (2.9)	
**Focus**			<.001
	Guideline (n=5)	11.2 (10.0)	
	Advice (n=80)	1.3 (2.1)	
	News (n=152)	2.3 (3.1)	
	Advertisement (n=65)	0.7 (1.4)	
	Opinion (n=7)	0.9 (0.9)	
	Insurance or health care cost (n=2)	1.5 (2.1)	
	Other IPF^c^-related focus (n=131)	1.7 (4.6)	
	Non-IPF–related focus (n=81)	0.1 (0.3)	

^a^Maximum total score of 30.

^b^Calculated using the χ^2^ test.

^c^IPF: idiopathic pulmonary fibrosis.

**Table 3:** Variables associated with content score in individual posts on adjusted analysis.^a^

Variables			Coefficient (95% CI)	OR^b^ or IRR^c^ (95% CI)	*P* value
**Zero-inflation model variables^d^**					
	**Post focus**				
		Guideline	–6.58 (–10.21 to –2.95)	0.00139 (0.0000368-0.0523)	<.001
		Non-IPF–related	25.68 (19.60, 31.77)	1.42×10^11^ (3.25×10^8^- 6.27×10^13^)	<.001
	Age of post (days)		0.00189 (0.000493 to 0.00329)	1.002 (1.0005-1.003)	.008
**Count model variables^e^**					
	Number of comments		0.0496 (0.00738 to 0.0918)	1.05 (1.01-1.10)	.02
	Industry post source		–2.55 (–2.84 to –2.26)	0.0783 (0.0586-0.104)	<.001
	**Post focus**				
		Guideline	Comparator	Comparator	N/A^f^
		Other IPF-related	–1.41 (–1.92 to –0.89)	0.245 (0.146-0.411)	<.001
		Non-IPF-related	–3.18 (–4.44 to –1.93)	0.0415 (0.0118-0.146)	<.001
	Age of post (days)		0.000213 (0.0000392 to 0.000387)	1.0002 (1.00004-1.0004)	.02
	Presence of link		1.64 (1.09 to 2.20)	5.18 (2.96-9.04)	<.001

^a^Clustered according to Facebook page or group that the post was made in.

^b^OR: odds ratio (for zero-inflated model variables).

^c^IRR: incident rate ratio (for count variables).

^d^Original zero-inflated model included a trinomial variable for post source and a trinomial variable for post focus.

^e^Original count model included number of likes, number of shares, trinomial post source variable, and presence of video or picture in a post.

Although not identified as a prespecified hypothesis, posts containing an external link had a higher content score on both unadjusted and adjusted analyses (Table 3). Posts containing useful scientific information generally had lower viewer engagement. By contrast, posts that contained useful supportive content had higher viewer engagement.

### Harmful Post Content

Only 5% of the posts contained potentially harmful information, but 35% of all groups or pages contained such posts. Although we hypothesized that posts with higher content scores would be less likely to contain potentially harmful content, we found that posts with higher content scores were actually more likely to contain potentially harmful information on both unadjusted and adjusted analyses (Table S4 in Multimedia Appendix 1).

We also hypothesized that post source other than a foundation/medical organization or medical professional, post with a focus other than IPF guidelines, and posts with greater viewer engagement would be associated with higher odds of a post containing potentially harmful content. On unadjusted analysis, groups or pages with a nonmedical user source were more likely to contain potentially harmful content compared to other sources. On adjusted analysis, posts with an industry source were less likely to contain potentially harmful content, and no other sources had an association with posts containing potentially harmful content. On adjusted analysis, posts with a guideline focus were less likely to contain potentially harmful content (Table S4 in Multimedia Appendix 1).

With respect to viewer engagement, posts containing potentially harmful content had significantly fewer likes on unadjusted analysis. On adjusted analysis, posts with greater than 5 likes or greater than 5 comments had a lower likelihood of containing potentially harmful content. Number of post shares was not associated with potentially harmful content.

## Discussion

### Principal Findings

To our knowledge, this is the first study to assess the content and quality of information about IPF on Facebook. Facebook is the second most widely used social media platform in the United States [6], emphasizing the importance of evaluating the content of health-related information disseminated through this platform. Prior studies have assessed content of Facebook posts from groups or pages in other diseases [15-20], but few have assessed post quality, instead focusing on descriptive analyses. Assigning content scores to posts based on guideline recommendations is a novel method for analyzing health-related posts on Facebook.

Previous surveys have shown that patients with pulmonary fibrosis perceive a lack of available resources and information about their disease [2]. Other studies demonstrate that social media is perceived by patients as an important resource for medical information and dialogue with health professionals [15,21]. We found that most of the identified posts in our study were made by nonmedical users, with very few posts coming from foundations, industry, or medical professionals. The relatively few posts from medical professionals highlights an area for future initiatives aimed at improving access to reliable health-related information on social media for patients with IPF and their caregivers.

The most frequent foci of posts pertaining to IPF included comments on IPF-related news (29%), asking for or receiving advice (15%), and advertising (12%). The frequency of posts with an advertisement focus was lower in our study than reported in previous studies of other chronic diseases [16,18,19], which may represent the small number of commercially available treatments for IPF. We found that 20% of posts presented useful scientific information and 24% provided other useful forms of support (eg, providing information regarding IPF support group meetings). The percentage of useful posts in our study was higher than that reported in similar studies evaluating Facebook content for other chronic diseases [16,22]. This may be related to a tight-knit network of patients with IPF that engage on social media or could reflect our strict inclusion criteria that excluded evaluation of pages or groups more peripherally associated with IPF, although this requires further study.

We found a negative correlation between number of likes or comments and content score. This suggests that Facebook posts containing more useful content may generate less attention, similar to findings seen in IPF-related YouTube videos [4]. Finding ways to make posts with useful content more visible represents an important area for future research. Although relatively few posts (5%) contained potentially harmful information, one third of pages or groups contained posts with potentially harmful content. This is a lower rate of harmful IPF content than observed on YouTube and other internet resources [3,4].

We found an association between higher content score and posts containing potentially harmful information. This indicates that harmful information about IPF on Facebook is surrounded by useful guideline information, which likely makes it more challenging for patients to distinguish accurate from harmful information. A possible explanation for this association is the presence of old posts discussing historical management approaches that have more recently been disproven (eg, inhaled N-acetylcysteine) [23]. Our findings suggested less frequent harmful content from groups or pages with a nonmedical user source and in posts from an industry source or with a guideline focus. These findings could be used to help direct patients to posts that are less likely to contain harmful information, although more research in this area is required.

### Strengths and Limitations

A strength of our study was not restricting our analysis to the most recent posts; however, this required evaluating every 10th post for content to ensure feasibility. This reduced our sampling of less common post sources such as medical professional–generated content, and we may have missed encountering specific harmful interventions that are only rarely discussed. We also only included open or public groups in the English language, as we were unable to access closed groups or reliably translate posts made in other languages. It is unclear if inclusion of these closed or non-English groups or pages would have led to significantly different findings. Additionally, we only included pages or groups focused on IPF. If we examined pages or groups about any form of ILD, our sample size would have been greater, although less specific for IPF and guideline-related content.

### Conclusions

This study shows that there is useful information about IPF that is available to patients and their caregivers on Facebook. Despite these findings, patients lack clear instruction on how to distinguish between posts containing useful versus harmful information. This is further complicated by the fact that potentially harmful information is often paired alongside useful guideline content. Moving forward, health care professionals need to identify ways to help patients discriminate between useful and potentially harmful information presented on social media. Post focus or source may provide clues in this regard. Health care professionals should also strive to increase medical professional–generated content aimed at patient education about IPF on Facebook. Additionally, encouraging posts that contain useful information to generate increased viewer engagement (likes, shares, and comments) will be critical to enhancing the dissemination of accurate medical information on Facebook. 

## Appendix

Multimedia Appendix 1 Supplementary data: Tables S1-S4, Figure S1.

## Abbreviations

ILD: interstitial lung disease
IPF: idiopathic pulmonary fibrosis
