# Supplementary material for: Social Media Content of Idiopathic Pulmonary Fibrosis Groups and Pages on Facebook: Cross-sectional Analysis
Source: JMIR Public Health Surveill. 2021 May 31;7(5):e24199. doi: 10.2196/24199 (PMC8204239; doi:10.2196/24199)
Supplement: Multimedia Appendix 1 [file publichealth_v7i5e24199_app1.docx]

**Table S1:** List of pre-specified hypotheses.

| **#** | **Hypothesis** |
| --- | --- |
| 1 | Higher content score in a post is associated with post source of foundation/medical organization or medical professional, IPF guideline focus, and greater viewer engagement. |
| 2 | Higher content score is associated with reduced odds of a post containing potentially harmful content. |
| 3 | Odds of a post containing potentially harmful content is positively associated with post source other than foundation/medical organization or medical professional, post focus other than IPF guideline, and greater viewer engagement. |

**Table S2:** Classification of guideline content

| **Category** | **Criteria** | **Examples** |
| --- | --- | --- |
| Definition  (/3) | Chronic lung disease  Scar tissue/fibrosis  Idiopathic | Chronic, progressive  Scarring, fibrosis  Idiopathic, unknown etiology/cause |
| Signs/ Symptoms  (/5) | Isolated pulmonary disorder  Dyspnea  Cough  Clubbing  Crackles | No symptoms outside lungs  Shortness of breath, breathing difficulty  Finger changes, curved nails  Velcro sounds on lung auscultation |
| Risk Factors  (/5) | Cigarette smoking  Male sex  Older age  Acid reflux  Genetics | Cigarettes, smoke  Heartburn  Family history, familial, hereditary |
| Evaluation  (/7) | Decreased FVC  Decreased D_L_CO  Pulmonary function testing  Computed tomography  Serology  Lung biopsy  Multi-disciplinary discussion | Decreased lung volumes  Decreased oxygen transfer  Breathing/lung function tests  CT scans, HRCT chest, lung imaging  Autoimmune disease testing  Lung tissue sample  Multidisciplinary conference, review with multiple specialists |
| Management | Nintedanib  Pirfenidone  Anti-acid therapy  Oxygen  Pulmonary rehab  Vaccinations  Lung transplant  Palliative care | Ofev  Esbriet  Proton pump inhibitors, reflux therapy  Lung rehab, exercise  End of life care, symptom management |
| Outcomes | Mean survival  Acute exacerbations | Survival 2-5yrs from diagnosis  Acute worsening, rapid progression |

**Table S3:** List of potentially harmful therapies

| **Non-Recommended Therapies** | **Number Times of Recommended in Posts** |
| --- | --- |
| Alpha-lipolic acid | 1 |
| Azathioprine | 3 |
| Baking soda | 1 |
| Benzodiazepines | 2 |
| Cannabis | 1 |
| Chinese herbal medications | 4 |
| Chlorophyll | 1 |
| Coenzyme Q | 1 |
| Cyclophosphamide | 1 |
| Glutathione | 1 |
| Herbal medications | 1 |
| Homeopathy | 1 |
| Ibuprofen | 1 |
| Inhaled N-Acetylcysteine | 1 |
| Inhaled thyroid hormone | 1 |
| Ketogenic diet | 1 |
| Liposomal vitamin C | 1 |
| MALVA-H | 1 |
| Metformin | 2 |
| Mycophenolate mofetil | 1 |
| N-Acetylcysteine | 4 |
| Oil of oregano | 1 |
| Paleo diet | 1 |
| Pineapple juice & “juicing” | 2 |
| Corticosteroids | 4 |
| Stem cell therapy | 3 |
| Tyrosine kinase inhibitors | 1 |
| Uridine | 1 |
| Vitamin D | 1 |

**Figure S1:** Percentage of posts containing guideline recommended content (A), useful scientific info (B) and useful support (C).

1. ****
2. ****
3. ****

**Table S4:** Multivariable logistic regression for associations with the presence of potentially harmful content in a post.

| **Logistic model variables** | **Coefficient (95% CI)** | **OR (95% CI)** | **p-value** |
| --- | --- | --- | --- |
| **Association between content score and presence of potentially harmful content in post** | | | |
| **Content score** | 0.09 (0.04, 0.14) | 1.10 (1.04, 1.15) | **0.001** |
| **Age of post (days)** | 1.27*10^-4^ (-2.59*10^-4^, 5.13*10^-4^) | 1.0001 (0.9997, 1.0005) | 0.518 |
| **Post focus (trinomial variable)**  Guideline  Other IPF-related  Non-IPF-related | Comparator  12.91 (11.32, 14.50)  12.69 (10.75, 14.62) | Comparator  4.05*10^5^ (8.25*10^4^, 1.99*10^6^)  3.24*10^5^ (4.68*10^4^, 2.24*10^6^) | **<0.001**  **<0.001** |
| **Association between number of post likes and presence of potentially harmful content in post** | | | |
| **Likes (trinomial variable)**  0 likes  1-5 likes  >5 likes | Comparator  -0.44 (-1.32, 0.45)  -1.56 (-3.10, -0.02) | Comparator  0.64 (0.27, 1.57)  0.21 (0.05, 0.98) | 0.332  **0.048** |
| **Age of post (days)** | -2.08*10^-5^ (-3.77*10^-4^, 3.35*10^-4^) | 0.99998 (0.99962, 1.00034) | 0.909 |
| **Post focus (trinomial variable)**  Guideline  Other IPF-related  Non-IPF-related | Comparator  12.51 (11.36, 13.65)  12.16 (10.67, 13.65) | Comparator  2.71*10^5^ (8.58*10^4^, 8.47*10^5^)  1.91*10^5^ (4.30*10^5^, 8.47*10^5^) | **<0.001**  **<0.001** |
| **Association between number of post comments and presence of potentially harmful content in post** | | | |
| **Comments (trinomial variable)**  0 comments  1-5 comments  >5 comments | Comparator  0.40 (-0.34, 1.13)  -14.49 (-15.36, -13.62) | Comparator  1.48 (0.72, 3.08)  5.08*10^-7^ (2.13*10^-7^, 1.21*10^-6^) | 0.289  **<0.001** |
| **Age of post (days)** | 1.22*10^-4^ (-2.62*10^-4^, 5.07*10^-4^) | 1.0001 (0.9997, 1.0005) | 0.533 |
| **Post focus (trinomial variable)**  Guideline  Other IPF-related  Non-IPF-related | Comparator  14.37 (13.39, 15.36)  13.88 (12.87, 14.90) | Comparator  1.74*10^6^ (6.53*10^5^, 4.68*10^6^)  1.07*10^6^ (3.90*10^5^, 2.95*10^6^) | **<0.001**  **<0.001** |
| **Association between number of post shares and presence of potentially harmful content in post** | | | |
| **Shares (trinomial variable)**  0 shares  1-5 shares  >5 shares | Comparator  0.16 (-0.77, 1.09)  -1.08 (-3.27, 1.12) | Comparator  1.18 (0.47, 2.98)  0.34 (0.04, 3.06) | 0.731  0.336 |
| **Age of post (days)** | 1.06*10^-4^ (-2.69*10^-4^, 4.80*10^-4^) | 1.0001 (0.9997, 1.0005) | 0.580 |
| **Post focus (trinomial variable)**  Guideline  Other IPF-related  Non-IPF-related | Comparator  13.03 (11.92, 14.14)  12.56 (11.09, 14.04) | Comparator  4.56*10^5^ (1.51*10^5^, 1.38*10^6^)  2.85*10^5^ (6.49*10^4^, 1.25*10^6^) | **<0.001**  **<0.001** |
| **Association between post source and presence of potentially harmful content in post** | | | |
| **Post source**  Foundation/Medical professional  Industry/For profit  User | -0.43 (-2.25, 1.39)  -13.82 (-15.82, -11.82)  Omitted due to collinearity | 0.65 (0.10, 4.01)  9.94*10^-7^ (1.34*10^-7^, 7.38*10^-6^)  Omitted due to collinearity | 0.641  **<0.001**  Omitted |
| **Age of post (days)** | 6.75*10^-5^ (-3.09*10^-4^, 4.44*10^-4^) | 1.0001 (0.9997, 1.0004) | 0.725 |
| **Post focus (trinomial variable)**  Guideline  Other IPF-related  Non-IPF-related | Comparator  13.89 (12.63, 15.16)  13.43 (11.92, 14.93) | Comparator  1.08*10^6^ (3.06*10^5^, 3.82*10^6^)  6.77*10^5^ (1.50*10^5^, 3.06*10^6^) | **<0.001**  **<0.001** |
| **Association between IPF guideline focus and presence of potentially harmful content in post** | | | |
| **Guideline focus** | -14.83 (-15.94, -13.72) | 3.62*10^-7^ (1.20*10^-7^, 1.10*10^-6^) | **<0.001** |
| **Age of post (days)** | 6.66*10^-5^ (-3.07*10^-4^, 4.41*10^-4^) | 1.0001 (0.9997, 1.0004) | 0.727 |
| **Post source**  Foundation or medical professional  Industry/For profit  User | Comparator  -14.36 (-16.98, -11.74)  0.42 (-1.43, 2.27) | Comparator  5.79*10^-7^ (4.23*10^-8^, 7.94*10^-6^)  1.52 (0.24, 9.68) | **<0.001**  0.657 |
